# Prevalence, Genetic Diversity, and Temporary Shifts of Inducible Clindamycin Resistance *Staphylococcus aureus* Clones in Tehran, Iran: A Molecular–Epidemiological Analysis From 2013 to 2018

**DOI:** 10.3389/fmicb.2020.00663

**Published:** 2020-04-30

**Authors:** Mehdi Goudarzi, Nobumichi Kobayashi, Masoud Dadashi, Roman Pantůček, Mohammad Javad Nasiri, Maryam Fazeli, Ramin Pouriran, Hossein Goudarzi, Mirmohammad Miri, Anahita Amirpour, Sima Sadat Seyedjavadi

**Affiliations:** ^1^Department of Microbiology, School of Medicine, Shahid Beheshti University of Medical Sciences, Tehran, Iran; ^2^Department of Hygiene, School of Medicine, Sapporo Medical University, Sapporo, Japan; ^3^Department of Microbiology, School of Medicine, Alborz University of Medical Sciences, Karaj, Iran; ^4^Department of Experimental Biology, Faculty of Science, Masaryk University, Brno, Czechia; ^5^Department of Virology, Pasteur Institute of Iran, Tehran, Iran; ^6^School of Medicine, Shahid Beheshti University of Medical Sciences, Tehran, Iran; ^7^Department of Critical Care and Anesthesiology, Imam Hossein Hospital, Shahid Beheshti University of Medical Sciences, Tehran, Iran; ^8^Department of Internal Medicine, Shahid Beheshti University of Medical Sciences, Tehran, Iran; ^9^Department of Mycology, Pasteur Institute of Iran, Tehran, Iran

**Keywords:** methicillin-resistant *S*. *aureus*, methicillin-susceptible *S*. *aureus*, inducible resistance, staphylocoagulase, SCC*mec*, *agr* allotype, MLST

## Abstract

The prevalence of *Staphylococcus aureus* as an aggressive pathogen resistant to multiple antibiotics causing nosocomial and community-acquired infections is increasing with limited therapeutic options. Macrolide-lincosamide streptogramin B (MLSB) family of antibiotics represents an important alternative therapy for staphylococcal infections. This study was conducted over a period of five years from August 2013 to July 2018 to investigate the prevalence and molecular epidemiology in Iran of inducible resistance in *S. aureus*. In the current study, 126 inducible methicillin-resistant *S. aureus* (MRSA) (*n* = 106) and methicillin-sensitive *S. aureus* (MSSA) (*n* = 20) isolates were characterized by *in vitro* susceptibility analysis, resistance and virulence encoding gene distribution, phenotypic and genotypic analysis of biofilm formation, prophage typing, *S. aureus* protein A locus (*spa*) typing, staphylocoagulase (SC) typing, staphylococcal cassette chromosome *mec* (SCC*mec*) typing, and multilocus sequence typing. Of the 126 isolates, 76 (60.3%) were classified as hospital onset, and 50 (39.7%) were classified as community onset (CO). Biofilm formation was observed in 97 strains (77%). A total of 14 sequence types (STs), 26 *spa* types, 7 coagulase types, 9 prophage types, 3 *agr* types (no *agr* IV), and 9 clonal complexes (CCs) were identified in this study. The prevalence of the inducible MLSB (iMLSB) *S. aureus* increased from 7.5% (25/335) to 21.7% (38/175) during the study period. The iMLSB MRSA isolates were distributed in nine CCs, whereas the MSSA isolates were less diverse, which mainly belonged to CC22 (7.95%) and CC30 (7.95%). High-level mupirocin-resistant strains belonged to ST85-SCC*mec* IV/t008 (*n* = 4), ST5-SCC*mec* IV/t002 (*n* = 4), ST239-SCC*mec* III/t631 (*n* = 2), and ST8-SCC*mec* IV/t064 (*n* = 2) clones, whereas low-level mupirocin-resistant strains belonged to ST15-SCC*mec* IV/t084 (*n* = 5), ST239-SCC*mec* III/t860 (*n* = 3), and ST22-SCC*me*c IV/t790 (*n* = 3) clones. All the fusidic acid–resistant iMLSB isolates were MRSA and belonged to ST15-SCC*mec* IV/t084 (*n* = 2), ST239-SCC*mec* III/t030 (*n* = 2), ST1-SCC*mec* V/t6811 (*n* = 1), ST80-SCC*mec* IV/t044 (*n* = 1), and ST59-SCC*mec* IV/t437 (*n* = 1). The CC22 that was predominant in 2013–2014 (36% of the isolates) had almost disappeared in 2017–2018, being replaced by the CC8, which represented 39.5% of the 2017–2018 isolates. This is the first description of temporal shifts of iMLSB *S. aureus* isolates in Iran that identifies predominant clones and treatment options for iMLSB *S. aureus*–related infections.

## Introduction

*Staphylococcus aureus* is one of the most common aggressive pathogen that causes many diseases in humans and animals such as skin and soft tissue infections, osteomyelitis, bacteremia, and endocarditis (Gordon and Lowy, 2008). The expression of virulence factors promoting adhesion needs nutrients, and evasion of host immunologic responses including cell surface components (collagen-binding protein, clumping factor, fibronectin-binding protein, and elastin-binding protein), secreted factors (staphylokinase toxic shock syndrome toxin-1), hemolysin, exfoliative toxins (ETA and ETB), staphylococcal enterotoxins (SEs), and lipase and Panton–Valentine leukocidin (PVL), besides the presence of antibiotic resistance genes, turns *S. aureus* into a very pathogenic microorganism (Gordon and Lowy, 2008; Gould et al., 2012).

In addition to the aforementioned, the biofilm-forming of *S. aureus* strains can play a key role in pathogenesis and resistance to antimicrobials (Luther et al., 2018). The rate of infections due to *S. aureus*, especially the antibiotic-resistant strains, has dramatically increased recently, which is becoming a serious problem all over the world (Gould et al., 2012). Emerging simultaneous resistance to multiple antibacterial agents underscores the necessity for therapeutic alternatives for the treatment of bacterium-related infections (Pantosti et al., 2007; Dadashi et al., 2018). Although the use of effective antibiotics such as vancomycin, linezolid, and quinupristin–dalfopristin is considered appropriate for therapy, widespread utilization of these antibiotics has made the current usage of these therapeutic options often unsuccessful (Pantosti et al., 2007; Gould et al., 2012; Wang et al., 2012; Dadashi et al., 2018). In recent years, the use of macrolide–lincosamide–streptogramin group B (MLSB) antibiotics has been favored, which is regarded as an alternative approach to treating such infections (Patel et al., 2006). Clindamycin, a member of MLSB family, serves as one such effective therapeutic alternative for treating *S. aureus* infections, because of its proven efficacy, safety, convenience of administration (parenteral and oral), and excellent pharmacokinetic properties. However, one important issue in clindamycin administration is the potential emergence of inducible clindamycin resistance, which may increase the risk of clinical failure (Chavez-Bueno et al., 2005; Adhikari et al., 2017).

Recent published data indicate that there has been a concurrent worldwide increase in the prevalence of inducible clindamycin resistance in different areas (Abimanyu et al., 2012; Wang et al., 2012). Evidence of epidemiological researches has revealed that iMLSB *S. aureus* strains are mostly genetically distinct from each other. One of the most prevalent iMLSB *S. aureus* strains in the United States belongs to sequence type 8 (ST8) strains, whereas in European countries, the majority of iMLSB *S. aureus* strains circulating was affiliated with the clonal complexes CC5 and CC8 (Ilczyszyn et al., 2016). Many studies across the world have focused on molecular epidemiology and analysis of iMLSB *S. aureus* strains isolated from the clinic (Lewis and Jorgensen, 2005; Patel et al., 2006; Adhikari et al., 2017). In our country, data on the prevalence or genetic diversity of inducible clindamycin resistance among *S. aureus* are very unknown, and there have been no published data on the molecular epidemiological characterization of the inducible clindamycin-resistant *S. aureus* strains in Iran. Here we provide molecular characterization of iMLSB *S. aureus* strains. With this aim, phenotypic and genotypic resistance patterns, presence of different classes of prophages, biofilm-forming ability, presence of the *icaABCD*, adhesion genes, and virulence factors were assessed. Then, multilocus sequence typing (MLST), staphylococcal cassette chromosome *mec* (SCC*mec*), *agr*, *coa*, and *spa* typing methods were used to characterize the genotype of the iMLSB *S. aureus* strains. To the best of our knowledge, this is the first report on the molecular characterization of the inducible clindamycin-resistant *S. aureus* strains from Iran.

## Materials and Methods

### Study Population, Bacterial Isolation, and Inducible Clindamycin Resistance Screening

A total of 1,161 non-duplicated clinical *S. aureus* isolates were obtained from different clinical specimens including wound, blood, pus, urine, sputum, conjunctivitis, and body fluids from both genders and all age groups of patients, over a period of 5 years from August 2013 to July 2018. This cross-sectional study was conducted in four hospitals affiliated to Shahid Beheshti University of Medical Sciences (Loghman, Shohada, Taleghani, Emam Hossein). The processing of all samples was done in 2 h. Among these isolates, 126 *S. aureus* isolates were identified with iMLSB phenotype based on the routine biochemical techniques such as Gram staining; colony morphology comprising shape, size, color, and hemolysis patterns; catalase test; tube coagulase test; growth on mannitol salt agar; and DNase. Polymerase chain reaction (PCR) assay targeting the *S. aureus*–specific *nuc* gene was applied to verify the isolates (Goudarzi et al., 2016b). *Staphylococcus aureus* strains were studied to identify inducible resistance phenotypes according to the Clinical and Laboratory Standard Institute (CLSI) D-zone test. The D-zone test was performed by placing 15 μg erythromycin and 2 μg clindamycin disks at a spaced 15 to 26 mm apart from center to center on the Mueller–Hinton agar (Merck, Darmstadt, Germany) plate inoculated with a 0.5 McFarland-equivalent bacterial suspension. The results also were read at 16 to 18 h incubation at 37°C and ambient air, using transmitted and reflected light; inducible clindamycin resistance was verified if the clindamycin zone of inhibition adjacent to the erythromycin disk (D-shape) was flattened. A confirmatory microdilution broth test was done on all isolates for further confirmation. Briefly, any growth in the same well that contained 4 μg/mL erythromycin and 0.5 μg/mL clindamycin was set as a positive test and vice versa. *Staphylococcus aureus* ATCC 25923 was used to perform routine quality control of antibiotic disks. Confirmed isolates were kept into tryptic soy broth (TSB; Merck) with 20% glycerol at −70°C for molecular testing.

Hospital-onset (HO) *S. aureus* was set if the positive culture of *S. aureus* was obtained on or after 96 h of admission to a hospital. Community-onset (CO) *S. aureus* was set if the culture was obtained prior to 4 days of hospitalization with one or more of the following criteria: (1) a history of hospitalization, surgery, dialysis, or residence in a long-term care facility in 12 months prior to culture date or (2) the presence of a central vascular catheter within 2 days before *S. aureus* culture. Invasive *S. aureus* infection was defined according to the Centers for Disease Control and Prevention defining isolation of *S. aureus* from typically sterile body sites such as the blood, bone, fluids (pericardial, joint/synovial, peritoneal, cerebrospinal, and pleural), internal body sites (brain, lymph node, pancreas, ovary, liver, spleen, heart, and kidney), or other normally sterile sites (Goudarzi et al., 2016a). The Ethics Committee of the Shahid Beheshti University of Medical Sciences in Tehran, Iran, certified the protocol of this project (IR.SBMU. MSP.REC.1396.412).

### Determining Resistance Pattern

Phenotypic methicillin resistance screening was performed by placing the cefoxitin disk (30 μg) on Mueller–Hinton agar (Merck), previously inoculated with a 0.5 McFarland-equivalent bacterial suspension. *In vitro* susceptibility of the isolates to kanamycin, ciprofloxacin, penicillin, quinupristin–dalfopristin, rifampin, tetracycline, linezolid, teicoplanin, amikacin, tobramycin, gentamicin, and trimethoprim–sulfamethoxazole (Mast; Merseyside, United Kingdom) was done according to the modified Kirby–Bauer disk diffusion method on Mueller–Hinton agar plates, as per CLSI recommendation. The European Committee for Antimicrobial Susceptibility Testing (EUCAST) breakpoint was used for interpreting the results of fusidic acid and ceftriaxone disks. As specified by the CLSI guidelines, the broth microdilution method was applied for determining the minimum inhibitory concentration (MIC) of vancomycin and mupirocin. Minimum inhibitory concentration values of 8 to 256 and ≥512 μg/mL were established to show low-level and high-level mupirocin resistance (LLMUPR, HLMUPR) of the strains, in the respective order. As specified by the CLSI guidelines, MIC breakpoints for vancomycin were set as follows: susceptible, ≤2 μg/mL; intermediate, 4–8 μg/mL; and resistant, ≥16 μg/mL. Fusidic acid MICs were specified by the broth microdilution method and interpreted based on EUCAST guidelines. Minimum inhibitory concentration breakpoints were set as follows: susceptible, ≤1 μg/mL; and resistant, >1 μg/mL. The *S. aureus* ATCC 25923 and ATCC 29213 strains were put to test as control strains. Sigma Chemical Co. (St. Louis, MO, United States) provided the study with the powders of antibiotics.

### DNA Extraction

To extract DNA, overnight cultures of *S. aureus* strains on 5% sheep blood agar (Merck) were used by applying the InstaGene Matrix kit (Bio-Rad, Hercules, CA, United States) following the manufacturer’s instructions and adding lysostaphin (Sigma-Aldrich; St. Louis, United States) for bacterial lysis. Gel electrophoresis and NanoDrop 2000 spectrophotometer (Thermo, Wilmington, Delaware, United States) were applied, respectively, to test the quality and quantity of isolated *S. aureus* DNA (Goudarzi et al., 2016a, b). Seemingly, if the purity were appropriated, it would be applied as the template for PCR. Two hundred microliters of elution buffer [10 mM Tris–Cl, 0.5 mM EDTA (pH 9.0)] was applied to elute the extracted DNA, which was then kept at −20°C until use.

### Detecting Antimicrobial Resistance Determinants and Virulence Factors

Polymerase chain reaction method was used for genotypic amplification of the *mecA* gene (Goudarzi et al., 2016a, b). Isolates exhibiting phenotypic resistance to particular antimicrobial agents were put to test for the presence of resistance genes [*mecA*, *vanA*, *vanB*, *mupB*, *mupA*, *fusA*, *fusB*, *fusC*, *erm*(A), *erm*(B), *erm*(C), *tet*(M), *ant* (4′)*-Ia*, *aac* (6′)*-Ie/aph* (2″), *aph* (3′)*-IIIa*] (Castanheira et al., 2010a; Nezhad et al., 2017). Virulence factors involved SE genes (*sea*, *seb*, *etb*, *sec*, *sed*, *see*, *seg*, *seh*, *sei*, and *sej*) (Monday and Bohach, 1999), exfoliative toxin genes (*eta* and *etb*), Panton–Valentine leukotoxin genes lukS-PV and lukF-PV (*pvl*), and toxic shock syndrome toxin genes (*tst*) (Goudarzi et al., 2016a; Nezhad et al., 2017).

### Phenotypic Analysis of Biofilm Formation

Slime production assay or Congo red agar (CRA) method and microtiter plate (MtP) assay were recruited as *in vitro* methods for the detection of phenotypic biofilm formation. In CRA method, briefly, after preparation of CRA by adding 0.8 g of CR (Sigma-Aldrich; St. Louis, United States) to 1 L of brain heart infusion agar (Merck) and autoclaving it, filters used for add saccharose (36 g) (Sigma-Aldrich; St. Louis, United States) to CRA. Bacteria were inoculated on CRA and incubated at 37°C for 24 h and then overnight at room temperature. Biofilm formation was categorized in four levels based on colony color that strains appeared: (i) strong biofilm producer strains (very black colonies), (ii) moderate biofilm producer strains (black colonies), (iii) weak biofilm producer strains (gray colonies), and (iv) biofilm non-producer strains (red colonies) (Arciola et al., 2002; Yousefi et al., 2016).

The MtP assay as a quantitative method for biofilm detection was carried out as described previously. Concisely, an overnight culture of bacterial isolates in TSB (Merck) containing 1% glucose was diluted to 1:100 with fresh medium. Sterile MtP with flat-bottomed 96-well polystyrene was filled with 200 μL of the diluted culture and incubated at 37°C for 24 h. After incubation, wells were washed three times with 200 μL of phosphate-buffered saline (pH 7.2) to remove planktonic bacteria. Afterward, wells were fixed by 99% methanol, dried at room temperature, and then stained with 0.1% safranin. Safranin dye bound to the adherent cells was dissolved with 1 mL of 95% ethanol per well. As a negative control, 200 μL of TSB–1% glucose was used. The optical density (OD) of each well was measured using an enzyme-linked immunosorbent assay reader at a wavelength of 490 nm. Optical density cutoff defined as average OD of negative control + 3 × standard deviation of the negative control. Biofilm formation of strains was analyzed according to the absorbance of the safranin-stained attached cells and interpreted as per the criteria described by Stepanović et al. (2007). Accordingly, the degree of biofilm production was categorized into strong, moderate, weak, or without biofilm. For quality control, *Staphylococcus epidermidis* ATCC 35984 strain was used in each run.

### Genetic Analysis of Biofilm Formation

Polymerase chain reaction assays for the detection of *icaABCD*, *cna*, *ebp*, *fnbB*, *fnbA*, *clfB*, *clfA*, and *bap* genes were performed as described previously (Nemati et al., 2009; Yousefi et al., 2016). Detection of arginine catabolic mobile elements for definitive confirmation of USA300 was performed as previously described by Diep et al. (2008).

### Prophage Typing

iMLSB-positive *S. aureus* were characterized by prophage typing by using multiplex-PCR assay and oligonucleotide primers SGA, SGB, SGF (SGFa and SGFb), SGD, and SGL prophages as explained previously by Pantůček et al. (2004) and Rahimi et al. (2012).

### *Staphylococcus aureus* Protein A Locus (*spa*) Typing

*Staphylococcus aureus* isolates underwent *spa* typing as recommended by Harmsen et al. (2003); all PCR products were Sanger sequenced in both directions. The sequences obtained were edited using the Chromas software (version 1.45; Technelysium, Tewantin, Australia). Ridom SpaServer database^1^ was applied in order to assign the *spa* type.

### Staphylocoagulase Typing

Four sets of multiplex PCR reactions were used for assigning staphylocoagulase (SC) types (I–X) according to the procedure of Hirose et al. (2010). Set A contained primers for identifying SC types I, II, III, IVa, IVb, Va, and VI, whereas set B contained primers for identifying SC types VII, VIII, and X. Set 3 was used for identifying SC types IX and Vb. SC types IVa and IVb were distinguished using a set of four primers.

### SCC*mec* Typing

The multiplex PCR amplification was done with specific primers for SCC*mec* typing, as recommended by Boye et al. (2007). The prototype strains applied as control strains for typing were *S. aureus* ATCC 10442 (type I), *S. aureus* N315 (type II), *S. aureus* 85/2082 (type III), *S. aureus* MW2 (type IV), *S. aureus* WIS 173 (type V), and *S. aureus* HDE288 (type VI).

### *agr* Typing

Multiplex PCR was performed for *agr*-type detection using primer set comprising a common forward primer (pan-agr) and reverse primers (*agr*1, *agr*2, *agr*3, and *agr*4) specific to each *agr* group (Gilot et al., 2002).

### Multilocus Sequence Typing

All the 126 *S. aureus* isolates with iMLSB phenotype were further characterized by MLST as described by Enright et al. (2000) by sequencing an internal fragment of seven unlinked housekeeping genes to identify the following allelic profiles: phosphate acetyltransferase (*pta*), carbamate kinase (*arcC*), triosephosphate isomerase (*tpi*), shikimate dehydrogenase (*aroE*), guanylate kinase (*gmk*), acetyl-coenzyme A acetyltransferase (*yqiL*), and glycerol kinase (*glp*). The purification of PCR products was done by performing the Qiagen PCR purification kit. In addition, the two strands were sequenced on the ABI Prism 377 automated sequencer (Applied Biosystems, Perkin-Elmer Co., Foster City, CA, United States). Finally, STs were submitted to the online MLST website through the submission of DNA sequences^2^.

### Ethics Statement

The current study protocol was approved by the Ethics Committee of the Shahid Beheshti University of Medical Sciences in Tehran, Iran (IR.SBMU.MSP.REC.1396.700). Written informed consent was obtained from participants.

## Results

### Isolation and Identification of *S. aureus*

A total of 126 *S. aureus* isolates with inducible resistance phenotype were identified, including 106 methicillin-resistant *S. aureus* (MRSA) and 20 methicillin-sensitive *S. aureus* (MSSA) isolates representing 84.1 and 15.9% of isolates, respectively. Precisely, of 126 iMLSB *S. aureus* isolates, 41 were collected from female patients (32.6%), and the rest were collected from male patients (85, 67.4%). In the present study, 43 strains (34.1%) were isolated from wound, 32 (25.4%) from blood, 15 (11.9%) from body fluids, 14 (11.1%) from pus, 12 (9.5%) from urine, 7 (5.6%) from sputum, and 3 (2.4%) from conjunctiva. Of the 126 isolates, 76 (60.3%) were classified as HO, and 50 (39.7%) were classified as CO. According to the case notes, the rates of invasive and non-invasive *S. aureus* with iMLSB phenotype were found to be 37.3 and 62.7%, respectively. The patients’ average age was 38 years. The patients were distributed in four age groups: 21 patients ≤20 years (16.7%), 60 patients between 21 and 45 years (47.6%), 30 patients between 46 and 65 years (23.8%), and 15 patients ≥65 years (11.9%). Regarding the occurrence of inducible resistance in *S. aureus* strains, data exhibited that most cases belonged to the age groups of 21 to 45 years between 2013 and 2017, whereas in 2018, more than half of the cases were found to be in the age group between 46 and 65 years. Patients with invasive infections and HO were older. Of 21 patients aged ≤20 years, 12 (57.1%) of 60 patients were between 21 and 45 years, 28 (46.7%) of 30 patients were between 46 and 65 years, and 9 (30%) had CO infections. However, among 15 patients aged ≥65 years, only 1 (6.7%) had CO infections. There were also trends toward an increasing incidence of HO infections in elderly patients.

### Antimicrobial Susceptibility

The highest and lowest rates of resistance in 126 *S. aureus* isolates tested were related to penicillin (91.3%) and fusidic acid (5.6%), respectively. The entire strains were susceptible to teicoplanin, linezolid, and vancomycin. Resistance to tested antibiotics was higher in MRSA than in MSSA. The rate of resistance to the tested antibiotics, with the exception of ciprofloxacin, was higher among HO *S. aureus* strains across CO *S. aureus* strains. The frequency of resistance rate among MRSA and MSSA strains to antimicrobial agents is presented in Table 1.

**TABLE 1:** Antimicrobial resistance pattern of MRSA and MSSA isolates with iMLSB phenotype.

Antibiotic	106 MRSA isolates *n* (%)		20 MSSA isolates *n* (%)		Total*n*(%)
		
	Hospital onset	Community onset	Hospital onset	Community onset	
Penicillin	67(53.2)	39(31)	9(7.1)	−	115(91.3)
Gentamicin	76(60.3)	21(16.6)	5(4)	4(3.2)	106(84.1)
Tetracycline	45(35.7)	29(23)	9(7.1)	6(4.8)	89(70.6)
Kanamycin	34(27)	19(15.1)	8(6.3)	5(4)	66(52.4)
Ceftriaxone	41(32.5)	11(8.7)	7(5.6)	4(3.2)	63(50)
Amikacin	31(24.6)	17(13.5)	10(7.9)	1(0.8)	59(46.8)
Ciprofloxacin	23(18.2)	29(23)	5(4)	−	57(45.2)
Tobramycin	39(31)	5(4)	7(5.5)	−	51(40.5)
Rifampin	25(19.8)	9(7.1)	3(2.4)	2(1.6)	39(30.9)
Trimethoprim–sulfamethoxazole	17(13.5)	11(8.7)	5(4)	2(1.6)	35(27.8)
Quinupristin–dalfopristin	18(14.3)	9(7.1)	−	3(2.4)	30(23.8)
Mupirocin	13(10.3)	4(3.2)	5(4)	1(0.8)	23(18.3)
Fusidic acid	7(5.6)	−			7(5.6)

The entire *S. aureus* isolates were susceptible to vancomycin such that 15 isolates (11.9%) had MIC ≥ 0.5 μg/mL, 38 (30.2%) had MIC ≥ 1 μg/mL, and 73 (57.9%) exhibited MIC ≥ 2 μg/mL. The data of the microbroth dilution method illustrated that 23 isolates (18.3%) were mupirocin-resistant; of these, 11 (8.7%) and 12 (9.6%) were LLMUPR and HLMUPR, respectively. Of the 126 isolates of *S. aureus* with iMLSB phenotype, seven (5.6%) were fusidic acid-resistant. In total, 80.2% of isolates (101/126) were multidrug resistance (MDR), and the MDR rate for MRSA isolates (85.8%) was significantly higher than that for MSSA (50%). Information about simultaneous resistance patterns and distribution of clinical samples is presented in Table 2.

**TABLE 2:** Resistant pattern and distribution of samples in 126 *S. aureus* strains isolated from clinical sources.

Simultaneous resistance to antibiotics	Resistance profile	Resistance pattern^a^	Type of samples^b^ (n;%)	Number of isolates (%)
Nine	A	PG, GM, T, K, CIP, TN, SYN, RI, TS	W (6; 40), B (3; 20), BF (2; 13.3), U (4; 26.7),	15 (11.9)
Eight	B	PG, GM, T, K, CRO, AK, CIP, TN	W (9; 52.9), B (5; 29.4), P (3; 17.7)	17 (13.5)
	C	PG, GM, T, K, CRO, RI, TS, MUP	W (4; 33.3), B (3; 25), P (2; 16.7), BF (2; 16.7), S (1; 8.3)	12 (9.5)
	D	PG, GM, T, CRO, AK, RI, MUP, TS	W (3; 37.5), BF (1; 12.5), P (4; 50)	8 (6.3)
Seven	E	PG, GM, T, K, CRO, AK, CIP	W (4; 26.8), B (3; 20), BF (2; 13.3), U (2; 13.3), P (2; 13.3), S (2; 13.3)	15 (11.9)
	F	PG, GM, T, CRO, AK, TN, FC	B (5; 100)	5 (4)
Six	G	PG, GM, T, AK, CIP, SYN	W (5; 50), BF (3; 30), U (2; 20)	10 (7.9)
Five	H	PG, GM, T, K, TN	W (3; 42.9), B (3; 42.9), C (1; 14.2)	7 (5.6)
	I	PG, GM, CRO, TN, SYN	BF (2; 40), C (1; 20), P (1; 20), U (1; 20)	5 (4)
	J	PG, GM, TN, MUP, FC	W (2; 100)	2 (1.6)
Four	K	PG, GM, AK, RI	B (2; 50), BF (1; 25), P (1; 25)	4 (3.2)
	L	PG, GM, CRO, MUP	C (1; 100)	1 (0.8)
Two	M	PG, GM	S (4; 80), U (1; 20)	5 (4)
One	N	PG	W (5; 55.6), B (4; 44.4)	9 (7.1)
Without	O	–	W (2; 18.2), B (4; 36.3), U (2; 18.2), P (1; 9.1), BF (2; 18.2)	11 (8.7)

*^*a*^PG, penicillin; CRO, ceftriaxone; GM, gentamicin; K, kanamycin; AK, amikacin; TN, tobramycin; T, Tetracycline; CIP, ciprofloxacin; TS, trimethoprim–sulfamethoxazole; RI, rifampicin; MUP, mupirocin; FC, fusidic acid; SYN, quinupristin–dalfopristin. ^*b*^B, blood; W, wound; P, pus; BF, body fluid; U, urine; S, sputum; C, conjunctiva.*

### *agr* and SCC*mec* Typing

By using *agr* typing method, all 126 *S. aureus* isolates with iMLSB phenotype could be typed *agr* I–III. Of these isolates tested, 77 (10 MSSA and 67 MRSA) were *agr* I, 20 (MRSA) were *agr* II, and 29 (10 MSSA and 19 MRSA) were *agr* III. Staphylococcal cassette chromosome *mec* typing for MRSA isolates indicated that type IV was the predominant SCC*mec* type (57.6%, 61/106), followed by SCC*mec* III (33%, 35/106), SCC*mec* V (7.5%, 8/106), and SCC*mec* II (1.9%, 2/106). In addition, no MRSA isolates harbored SCC*mec* type I.

### Determination of Adhesion and Biofilm Production Ability

**FIGURE 1:**
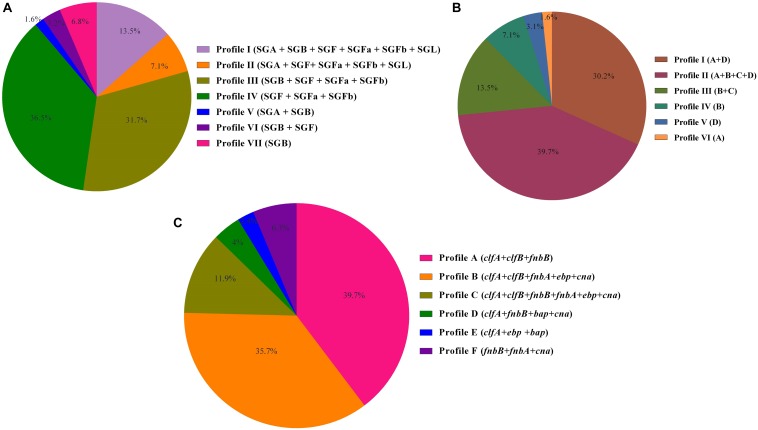
**(A)** Prophage profiles of 126 *Staphylococcus aureus* isolates with iMLSB phenotype. **(B)** *icaABCD* profiles of inducible clindamycin resistance *S. aureus*. **(C)** Adhesion profiles of *S. aureus* isolates investigated in this study.

Biofilm formation was observed in 97 strains (77%) using the CRA and MtP methods, whereas 29 strains (23%) were confirmed as non–biofilm producer strains. Of the total MRSA isolates, MtP detected 18 (17%) as weak, 23 (21.7%) as moderate, and 42 (39.6%) as strong biofilm producers. By CRA, 20 isolates (18.9%) were able to produce biofilm weakly, and 26 (24.5%) and 37 (34.9%) isolates presented moderate and potent biofilm formation, respectively. In MSSA isolates, MtP indicated weak biofilm production in five isolates (25), moderate biofilm production in three (15%), and strong biofilm production in six isolates (30%), whereas CRA detected four isolates (20%) as weak, five (25%) as moderate, and five (25%) as strong biofilm producers. The analysis of *icaA-D* genes among tested *S. aureus* strains showed that the most frequently detected gene was *icaD* (*n* = 92, 73%), followed by *icaA* (*n* = 90, 71.4%), *icaB* (*n* = 76, 60.3%), and *icaC* (*n* = 67, 53.2%). Overall, six different biofilm patterns were identified. Figure 1 presents the distribution of *icaABCD* among tested strains. Regarding the presence of adhesion genes, the seven most frequent adhesion-related genes were *clfA* (93.7%, 118/126) followed by *clfB* (87.3%, 110/126), *fnbB* (61.9%, 78/126), *cna* (57.9%, 73/126), *fnbA* (54%, 68/126), *ebp* (50%, 63/126), and *bap* (3.2%, 8/126), respectively. The adhesion profiles are shown in Figure 1. None of the MSSA strains carried the *bap* gene. In MSSA strains, three different adhesion patterns including *clfA* + *clfB* + *fnbB* (50%, 10/20), *clfA* + *clfB* + *fnbB* + *fnbA* + *ebp* + *cna* (25%, 5/20), and *fnbB* + *fnbA* + *cna* (25%, 5/20) were observed. Apparently, higher diversity among the adhesion patterns from MRSA (six patterns) than those from MSSA (three patterns) was noticed.

### Determination of Prophage Types and Toxin and Enterotoxin Encoding Genes

A total of 103 strains (81.7%) of the 126 inducible resistant *S. aureus* strains tested had one or more virulence genes. The most frequently attained virulence genes were *pvl* (23.8%, 30/126), followed by *sea* (19.8%, 25/126), *tst* (19%, 24/126), *sec* (11.1%, 14/126), *sed* (9.5%, 12/126), *seg* (4.8%, 6/126), *seb* (4%, 5/126), *see* (3.2%, 4/126), and *eta* (2.4%, 3/126), but *she*, *sei*, and *sej* encoding genes were detected in any of the total number of 126 isolates tested. In this analysis, six prophage types were grouped into seven separate prophage profiles. Figure 1 provides a summary data of the number and frequency of prophage profiles among isolates tested.

The data for antibiotic resistance genes in *S. aureus* strains studied revealed that the most common gene was *mecA* in 106 strains (84.1%), followed by *erm*(A) in 43 strains (34.1%), *erm*(B) in 41 (32.5%), *erm*(C) in 361 (28.6%), *ant* (4′)*-Ia* in 35 (27.8%), *tet*(M) in 32 (25.4%), *aac* (6′)*-Ie/aph* (2′′) in 29 (23%), *aph* (3′)*-IIIa* in 17 (13.5%), *mupA* in 12 (9.5%), *fusB* in 6 (4.8%) strains, and *fusC* in 1 strain. *vanB*, *mupB*, and *fusA* genes were not detected in none of experimented isolates.

### SC Typing

In the present study, all isolates could be typed by SC typing method. These isolates were distinguished into seven SC types (I–VI). The predominant SC type was III and included 56 isolates (44.4%), followed by II (28.6%, 36/126), IVa (9.5%, 12/126), VI (7.9%, 10/126), I (4%, 5/126), V (4%, 5/126), and IVb (1.6%, 2/126). A total of seven SC types and two types (II and IVa) were detected in MSSA isolates.

### MLST and *spa* Typing

All the isolates were typed by *spa* and MLST. A total of 26 *spa* types were attained in the entire 126 *S. aureus*. The isolates identified as invasive *S. aureus* were assigned to particular t790, t002, t008, t064, t030, t037, and t631 *spa* types. For CO, the isolates were assigned to *spa* types t005, t1869, t019, t021, t10795, t6811, t002, t045, t008, t065, t030, and t037. Among MSSA isolates, the most common *spa* types were t021, t005, t1869, and t318 representing 35 (7/20), 25 (5/20), 25 (5/20), and 15% (3/20), and among MRSA isolates, the top four *spa* types were t037, t437, t030, and t008 representing 10.4 (11/106), 9.4 (10/106), 8.5 (9/106), and 7.5% (8/106) of isolates. Multilocus sequence typing identified 14 ST types in both MRSA and MSSA isolates, namely, ST22 (14.3%, 18/126), ST30 (11.9%, 15/126), ST1 (1.6%, 2/126), ST772 (4.8%, 6/126), ST8 (10.3%, 13/126), ST239 (23%, 29/126), ST585 (1.6%, 2/126), ST5 (5.63%, 7/126), ST225 (1.6%, 2/126), ST80 (9.5%, 12/126), ST59 (4.8%, 6/126), ST338 (3.2%, 4/126), ST15 (4%, 5/126), and ST45 (4%, 5/126), which were further grouped into nine CCs including CC1, 5, 8, 15, 22, 30, 45, 59, and 80. Among MSSA, CC22 and CC30 were the dominant clones, each clone with 50% (10/20) of the isolates being assigned to these genotypes, and all were recovered from non-invasive *S. aureus* and both CO (55%, 11/20) and HO (45%, 9/20) cases. CC8 (41.5%, 44/106), CC80 (11.3%, 124/106), and CC5 (8.5%, 9/106) were the top three CCs in MRSA isolates. Table 3 represents the analysis of *S. aureus* clones obtained from patients based on invasive and non-invasive infections.

**TABLE 3:** Analysis of *S. aureus* clones obtained from patients based on invasive and non-invasive infections.

Clonal complex (CC)	Molecular types	Invasive *S. aureus*		Non-invasive *S. aureus*		Total, *n* (%)
			
		Community onset, *n* (%)	Hospital onset, *n* (%)	Community onset, *n* (%)	Hospital onset, *n* (%)	
CC22	ST22-SCC*mec* IV/t852	0(0)	0(0)	0(0)	2(100)	2(1.6)
	ST22-SCC*mec* IV/t790	0(0)	3(60)	0(0)	2(40)	5(4)
	ST22-SCC*mec* IV/t005	0(0)	0(0)	0(0)	1(100)	1(0.7)
	ST22/t005	0(0)	0(0)	2(40)	3(60)	5(4)
	ST22/t1869	0(0)	0(0)	4(80)	1(20)	5(4)
CC30	ST30-SCC*mec* IV/t605	0(0)	0(0)	0(0)	2(100)	2(1.6)
	ST30-SCC*mec* IV/t019	0(0)	0(0)	3(100)	0(0)	3(2.4)
	ST30/t318	0(0)	0(0)	0(0)	3(100)	3(2.4)
	ST30/t021	0(0)	0(0)	5(71.4)	2(28.6)	7(5.5)
CC1	ST772-SCC*mec* IV/t10795	0(0)	0(0)	3(60)	2(40)	5(4)
	ST772-SCC*mec* IV/t657	0(0)	0(0)	0(0)	1(100)	1(0.7)
	ST1-SCC*mec* IV/t6811	0(0)	0(0)	1(50)	1(50)	2(1.6)
CC5	ST5-SCC*mec* IV/t002	2(28.6)	3(42.8)	1(14.3)	1(14.3)	7(5.5)
	ST225-SCC*mec* II/t045	0(0)	0(0)	1(50)	1(50)	2(1.6)
CC8	ST8-SCC*mec* IV/t008	1(12.5)	3(37.5)	4(50)	0(0)	8(6.3)
	ST8-SCC*mec* IV/t064	2(40)	2(40)	1(20)	0	5(4)
	ST585-SCC*mec* III/t713	0(0)	0(0)	0(0)	2(100)	2(1.6)
	ST239-SCC*mec* III/t030	0	4(44.4)	1(11.2)	4(44.4)	9(7.1)
	ST239-SCC*mec* III/t037	7(63.6)	1(9.1)	0(0)	3(27.3)	11(8.7)
	ST239-SCC*mec* III/t631	0(0)	1(25)	0(0)	3(75)	4(3.2)
	ST239-SCC*mec* III/t860	0(0)	0(0)	0(0)	3(100)	3(2.4)
	ST239-SCC*mec* III/t388	0(0)	0(0)	0(0)	2(100)	2(1.6)
CC80	ST80-SCC*mec* IV/t044	0(0)	6(75)	0(0)	2(25)	8(6.3)
	ST80-SCC*mec* IV/t131	0(0)	0(0)	2(50)	2(50)	4(3.2)
CC59	ST59-SCC*mec* IV/t437	3(50)	2(33.3)	0(0)	1(16.7)	6(4.8)
	ST338-SCC*mec* III/t437	0(0)	0(0)	0(0)	4(100)	4(3.2)
CC15	ST15-SCC*mec* IV/t084	2(40)	1(20)	0(0)	2(40)	5(4)
CC45	ST45-SCC*mec* IV/t038	4(80)	0(0)	1(20)	0(0)	5(4)

### Temporal Changes in Inducible Resistant *S. aureus* Genotypes

Table 4 gives information about the percentage of inducible resistant *S. aureus* strains assigned to each CC for each year between 2013 and 2018. The prevalence of inducible resistance *S. aureus* isolates elevated in the experiment from 19.8% in 2013–2014 to 30.2% in 2017–2018. Apart from 2013 to 2014 in which CC22 genotype was the most prevalent CC among inducible resistance isolates (36%, 9/25), in the remaining years of study (2014–2018), the CC8 isolates were the most predominant genotype. The CC8, as a predominate genotype, was at its most prevalent in 2016–2017 (41.6%, 10/24). Its prevalence increased during the study period from 20% in 2013–2014 (5/25) to 39.5% (15/38) in 2017–2018. By contrast, the prevalence rate of CC22 decreased from 36% in 2013–2014 to 0% in 2017–2018. The CC30, the third predominated CC among our clinical isolates, had its most prevalence in 2017–2018 (18.4%, 7/38). The prevalence of this type varied dramatically each year during 2013–2018; despite a decrease to 8% (2/25) in 2013–2014, a slight increase in the prevalence of this type was noted during 2014–2015 (9.5%, 2/21) and 2015–2016 (16.7%, 3/18), followed by an overall decrease to 4.2% (1/24) in 2016–2017. The prevalence of this type increased to 18.4% (7/38) in 2017–2018.

**TABLE 4:** Distribution of characterized *S. aureus* isolates with iMLSB phenotype by year.

*S. aureus* clones	2013–2014, *n* (%)	2014–2015, *n* (%)	2015–2016, *n* (%)	2016–2017, *n* (%)	2017–2018, *n* (%)	Total, *n* (%)
CC8	5(11.4)	8(18.2)	6(13.6)	10(22.7)	15(34.1)	44(34.9)
CC22	9(50)	5(27.8)	1(5.6)	3(16.6)	0(0)	18(14.3)
CC30	2(13.3)	2(13.3)	3(20)	1(6.7)	7(46.7)	15(11.9)
CC80	3(25)	0(0)	0(0)	4(33.3)	5(41.7)	12(9.5)
CC59	0(0)	1(10)	0(0)	2(20)	7(70)	10(7.9)
CC5	2(22.2)	3(33.4)	2(22.2)	1(11.1)	1(11.1)	9(7.1)
CC1	4(50)	2(25)	2(25)	0(0)	0(0)	8(6.4)
CC45	0(0)	0(0)	4(80)	1(20)	0(0)	5(4)
CC15	0(0)	0(0)	0(0)	2(40)	3(60)	5(4)
Total	25(19.8)	21(16.7)	18(14.3)	24(19)	38(30.2)	126(100)

The first CC45 isolates were found in 2015–2016. The prevalence of this genotype diminished during the study period from 22.2% (4/18) in 2015–2016 to 4.2% (1/24) in 2016–2017 apart from 2017–2018 when no CC45 was detected. The CC45 was the second predominant genotype in 2015–2016 (22.2%, 4/18). The CC15, absent during 2013–2016, was detected during 2016–2017 (8.3%, 2/24), but remained at relatively low prevalence (7.9%, 3/38) in 2017–2018.

The CC59 isolates were not detected between 2013–2014 and 2015–2016. The CC59 isolates were detected for the first time in 2014–2015 (4.8%, 1/21), and increased to 8.3% (2/24) in 2016–2017. This genotype was present at its greatest prevalence in 2017–2018, accounting for 18.4% (7/38) of inducible resistance strains. The prevalence of CC80 varied dramatically during the 5-year period, this genotype was not identified among any of the 2014–2015 and 2015–2016 tested strains. In 2013–2014, only 12% (3/25) of isolates exhibited this genotype. An increase in the prevalence of this genotype was noted between 2016 and 2017 to 16.7% (4/24), followed by a decline to 13.2% (5/38) in 2017–2018. The CC5 genotype was present in each year between 2013 and 2018. The highest prevalence of this genotype occurred in 2014–2015 (14.3%, 3/21), followed by 11.1% (2/18) between 2015 and 2016. Overall, the prevalence of this genotype diminished during the study period from 8% (2/25) in 2013–2014 to 2.6% (1/38) in 2017–2018. The CC1 isolates were not identified between 2016 and 2018. It was the third predominant genotype in 2013–2014, accounting for 16% (4/25) of examined isolates, but it dropped to 9.5% (2/21) in 2014–2015 and then indicated an increase in 2015–2016 (11.1%, 2/18).

### Observations on Inducible Clindamycin-Resistant *S. aureus* Clones

The molecular characteristics of the isolates related to each genotype of inducible resistance MRSA and MSSA are shown in Table 5. Meanwhile, the details of each clonal lineage are explained below.

**TABLE 5:** Phenotypic and genotypic typing data for inducible resistance MRSA (*n* = 106) and MSSA (*n* = 20) isolates studied in Tehran, Iran, between 2013 and 2018.

Typing category results (n)										

CC	Genotype					Antibiotic resistance				
					
	MRSA	MSSA	*spa*	*agr*	*coa*	Phenotype profiles^a^ (% indicated when not 100%)	Genotype (%)	Virulence genes (% indicated when not 100%)	Biofilm status^b^ (% indicated when not 100%)	Prophage profile^c^ (% indicated when not 100%)
22	ST22-MRSA-IV (8)		t852 (2), t790 (5)	I	III	A (14.2), B (42.9), D (42.9)	*mecA* (100), *ant (4*′*)-Ia* (28.6), *aac (6*′*)-Ie/aph (2*′′) (42.9), *erm(C)* (57.1), *erm(B)* (42.9)	*pvl* (28.6), *tst* (42.9), *sea* (14.2)	W (57.1), S (42.9)	I (28.6), IV (71.4)
			t005 (1)	I	IVa	H	*mecA*, *erm(A)*, *erm(B)*	*pvl*	S	I
		ST22-MSSA (10)	t005 (5), t1869 (5)	I	IVa	N (40), K (30), H (30)	*erm*(A) (50), *aac* (6′)*-Ie/aph* (2′′) (20), *ant* (4′)*-Ia* (30), *tet*(M) (50), *erm(B)* (50)	*sec* (30)	N (50), W (30), S (20)	III (50), IV (50)
30	ST30-MRSA-IV (5)		t605 (2), t019 (3)	III	II	A(40), B (20), E (40)	*mecA* (100), *erm*(C) (60), *aac (6*′*)-Ie/aph (2*′′) (40), *aph (3*′*)-IIIa* (20), *tet(M)* (20)	*pvl* (60), *sea* (40)	N (20), W (40), S (40)	I (60), V (40)
		ST30-MSSA (10)	t021 (7), t318 (3)	III	II	G (30), N (10), I (10), O (40), L (10)	*erm*(A) (10), *erm*(B) (20), *erm*(C) (50), *ant* (4′)*-Ia* (30), *aph (3*′*)-IIIa* (10), *erm(B)* (60)	*sed* (40), *pvl* (30)	N (10), W (20), M (30), S (40)	VI (10), VII (30), III (30), IV (30)
1	ST1-MRSA-IV (2)		t6811 (2)	III	IVb	E (50), F (50),	*mecA* (100), *erm(A)* (100), *fusc* (50),	*sec*	W	III
	ST772-MRSA-IV (6)		t10795 (5)	II	III	B (60), E (40),	*mecA* (100), *aac (6*′*)-Ie/aph (2*′′) (40), *aph (3*′*)-IIIa* (60), *tet*(M) (60), *erm(B)* (100)	*pvl* (60), *sea* (40)	N (20), W (20), S (60)	I (40), IV (60)
			t657 (1)	II	IVa	I	*mecA*, *erm*(C)	*sea* + *seb*	S	VII
8	ST8-MRSA-IV (13)		t008 (8), t064 (5)	I	III	C (30.8), D (23.1), O (38.5), M (7.6)	*mecA* (100), *mupA* (46.1), *aph (3*′*)-IIIa* (61.5), *erm(C)* (38.5), *tet*(M) (38.5), *erm(B)* (30.8)	*pvl* (69.2), *sed* (30.8)	N (23.1), W (38.4), M (23.1), S (15.4)	I (69.2), IV (30.8)
	ST239-MRSA-III (29)		t030 (9), t037 (11), t631 (4), t860 (3), t388 (2)	I	III	A (20.7), B (13.8), C (10.4), D (3.4), E (17.3), F (6.9), G (13.8), I (6.9), K (3.4), O (3.4)	*mecA* (100), *mupA* (6.9), *fusB* (6.9), *erm*(A) (51.7), *erm*(B) (17.3), *erm*(C) (44.8), *ant* (4′)*-Ia* (58.6), *aac (6*′*)-Ie/aph (2*′′) (37.9), *aph (3*′*)-IIIa* (6.9), *tet*(M) (41.3)	*tst* (48.3), *sea* (55.1), *sec* (17.3), *seg* (10.4), *eta* (10.4)	N (13.8), W (13.8), M (10.4), S (62)	III (41.4), IV (44.8), VII (13.8)
	ST585-MRSA-III (2)		t713 (2)	I	III	I (50), E (50)	*mecA* (100), *tet*(M) (50), *erm(C)* (50)	*see*	S	IV
5	ST5-MRSA-IV (7)		t002 (7)	II	II	C (42.9), D (14.2), H (42.9)	*mecA* (100), *mupA* (57.1), *erm*(A) (57.1), *tet*(M) (71.4)	*tst* (42.9), *sea* (42.9), *sed* + *seg* (42.9)	N (42.9), M (57.1)	IV
	ST225-MRSA-II (2)		t045 (2)	II	II	G	*mecA* (100), *ant* (4′)*-Ia* (100), *aac (6*′*)-Ie/aph (2*′′) (100), *erm(B)* (50)	*tst* (100), *sed* (50)	S	VI
80	ST80-MRSA-IV (12)		t044 (8), t131 (4)	III	II	B (50), E (25), F (8.3), M (16.7)	*mecA* (100), *fusB* (8.3), *ant* (4′)*-Ia* (41.7), *aac (6*′*)-Ie/aph (2*′′) (33.3), *erm(A)* (83.3)	*pvl* (33.3)	N (25), M (41.7), S (33.3)	III (58.4), VI (8.3), II (33.3)
59	ST59-MRSA-IV (6)		t437 (6)	I	VI	E (16.7), F (16.7), N (66.6)	*mecA* (100), *fusB* (16.7), *erm*(B) (33.3), *erm*(C) (66.7)	*seb* (66.7)	N (50), M (33.3), S (16.7)	III
	ST338-MRSA-III (4)		t437 (4)	I	VI	A (50), M (50)	*mecA* (100), *ant* (4′)*-Ia* (75), *aph (3*′*)-IIIa* (50), *erm(B)* (75)	*see* (50)	N (25), M (25), S (50)	III
15	ST15-MRSA-IV (5)		t084 (5)	II	I	C (40), J (40), O (20)	*mecA* (100), *fusB* (40), *erm(B)* (80)	*pvl* (100)	N (40), M (40), S (20)	II
45	ST45-MRSA-IV (5)		t038 (5)	I	V	A (80), G (20)	*mecA* (100), *erm*(A) (100), *aac* (6′)*-Ie/aph* (2′′) (60)	*tst* (40), *sec* (80)	N (40), M (60)	III (20), IV (80)

*^*a*^Phenotypic profile are presented in Table 2. ^*b*^W, weak producer; M, moderate producer; S, strong producer, N, non-producer. ^*c*^Prophage profiles are presented in Figure 1A.*

#### CC1

In this study, most of the isolates identified as CC1/ST772-MRSA-V were represented by *spa* type t10795 (83.3%, 5/6). Among the CC1/ST772-MRSA-V isolates, resistance to aminoglycosides encoded by *aac (6*′*)-Ie/aph (2*′′) and *aph (3*′*)-IIIa*, and tetracycline encoded by *tet(M)* was common. Three isolates (60%) were able to produce biofilm strongly. The finding indicated that *fnbB* gene was present in all of the CC1 isolates. *icaA* and *icaD* were the most prevalent biofilm-related genes. More than half of the isolates (60%) carried *pvl* genes. The one remaining CC1/ST772-MRSA-V isolate was assigned to *spa* type t657 and *coa* type IVa. This isolate exhibited resistance to multiple antibiotics and harbored resistance genes *mecA* and *erm*(C); meanwhile, enterotoxin genes *sea* + *seb* carried simultaneously. The CC1/ST1-MRSA-V isolates exhibited *spa* type, t6811. One isolate carried *fusc* and exhibited resistance to fusidic acid at MIC 32 μg/mL. MDR pattern was detected among these isolates. Both isolates were identified to be biofilm producers albeit weakly. Toxin gene differs from those detected in CC1/ST772-MRSA-V found among the CC1/ST1-MRSA-V isolates, namely, *sec.* All the isolates exhibited erythromycin resistance encoded by *erm*(C).

#### CC5

All CC5/ST5-MRSA-IV isolates were assigned to single *spa* type t002. This isolates carried the enterotoxin genes *sea* (42.9%, 3/7) and *sed* + *seg* (42.9%, 3/7) and the toxin *tst* (42.9%, 3/7). Among the CC5/ST5-MRSA-IV isolates, resistance to erythromycin encoded by *erm*(A), tetracycline encoded by *tet*(M), and mupirocin encoded by *mupA* was common. Five of HLMUPR strains, which harbored the *mupA* gene, belonged to this CC (41.7%, 5/12). Nearly 60% of all isolates that occurred in these clones showed moderate biofilm formation. The remaining 40% were not able to produce biofilm. The top three adhesion-related genes among CC5 isolates were *clfA* (100%, 9/9), *clfB* (77.8%, 7/9), *ebp* (75%, 6/9), and *cna* (75%, 6/9). Of nine CC5 isolates, *icaA* and *icaD* were found in seven isolates, whereas *icaB* and *icaC* were detected in six isolates. The other CC5 genotypes identified (CC5/ST225-MRSA-II) were assigned to *spa* type t045, which exhibited resistance to multiple antibiotics including aminoglycosides resistance encoded by *ant* (4′)*-Ia (2)* and *aac (6*′*)-Ie/aph (2*′′) genes. *tst* encoding gene was detected in all isolates. All CC5/ST225-MRSA-II showed strong biofilm formation. The predominant prophage profile was SGF + SGFa + SGFb.

#### CC8

In this study, CC8 was represented by ST8-MRSA-IV (29.6%, 13/44), ST239-MRSA-III (65.9%, 29/44), and ST585-MRSA-III (4.5%, 4/44). *spa* types t008 (61.5%, 8/13) and t064 (38.5%, 5/13) predominated among the CC8/ST8-MRSA-IV isolates. Among 13 ST8-MRSA-IV strains, 8 isolates harbored the *arcA* and *opp3AB* genes and confirmed as USA300. The majority of CC/ST8-MRSA-IV (USA300), as a major global epidemic clone that has been noticed for its rapidity dissemination within the community and hospitals isolates, exhibited resistance to aminoglycosides encoded by *aph (3*′*)-IIIa* (61.5%, 8/13). Resistance to erythromycin encoded by *erm*(C) (38.5%, 5/13) and tetracycline encoded by *tet*(M) (38.5%, 5/13) was also detected in this genotype. Six strains belonging to CC/ST8-MRSA-IV clone demonstrated the HLMUPR phenotype and carried *mupA* gene. The findings showed that the majority of the isolates (77%, 10/13) was able to produce biofilm, although at different intensities. Nine isolates in this clone harbored the *pvl* genes (69.2%); the rest of four isolates carried the *sed* gene (30.8%).

The majority of CC8/ST239-MRSA-III isolates were assigned to *spa* type t037 (37.9%, 11/29), followed by t030 (31.1%, 9/29), t631 (13.8%, 4/29), t860 (10.3%, 3/29), and t388 (6.9%, 2/29). Resistance to multiple antibiotics simultaneously was identified in this genotype. Fusidic acid resistance encoded by *fusc* (6.9%, 2/29); tetracycline resistance encoded by *tet*(K) (41.4%, 12/29); aminoglycoside resistance encoded by *ant* (4′)*-Ia* (58.6%, 17/29), *aac (6*′*)-Ie/aph (2*′′) (37.9%, 11/29), and *aph (3*′*)-IIIa* (6.9%, 2/29); and mupirocin resistance encoded by *mupA* (6.9%, 2/29) were also noted. Almost more than half of the isolates carried *sea* (55.2%, 16/29), 48.3% (14/29) carried *tst*, and 17.2% (5/29) carried *sec* only three isolates harboring *seg* gene. All three ST239-SCC*mec* III/t860 isolates carried *eta* and demonstrated the LLMUPR phenotype. The CC8/ST239-MRSA-III isolates displayed high levels of biofilm production (86.2%, 25/29). Regarding the biofilm-related genes, the *icaD* (70.5%, 31/44) was common, followed by *icaA* (68.2%, 30/44), *icaB* (68.2%, 30/44), and *icaC* (61.4%, 27/44). The top three adhesion-related genes among CC8 isolates were *clfA* (100%, 44/44), *clfB* (86.4%, 38/44), and *cna* (86.4%, 38/44).

Two remaining CC8/ST585-MRSA-III isolates were assigned to *spa* type t713 and carried multiple resistance genes, that is, *tet*(M), *erm*(C), and enterotoxin genes *see*. Both of these strains indicated strong biofilm formation. The majority of the CC8 isolates was found to harbor SGF + SGFa + SGFb proghages.

#### CC15

The five CC15/ST15 MRSA-IV isolates identified revealed the same *spa*, coa, *agr*, and prophage profile. Fewer than half of the isolates were resistant to fusidic acid encoded by *fusB*. All CC15/ST15 MRSA-IV isolates carried *pvl* genes. More than half of the isolates were biofilm producers. All CC15 isolates were positive for *fnbB* gene, and 80% of the isolates were found to carry all of the biofilm-related genes (*icaA*-*D*). One isolate was found to carry none of the tested *ica* genes. All of the CC15 isolates possessed SGA + SGF + SGFa + SGFb + SGL prophage profile.

#### CC22

Three *spa* types t852, t790, and t005 and two spa types t005, t1869 predominated among the CC22/ST22-MRSA-IV isolates (44.4%, 8/18) and CC22/ST22-MSSA (55.6%, 10/18) isolates. Among the MRSA isolates, resistance to erythromycin encoded by *erm(C)* and aminoglycosides encoded by *aac (6*′*)-Ie/aph (2*′′) were prevalent, whereas in MSSA isolates resistance to tetracycline encoded by *tet*(M) and that to aminoglycosides encoded by *ant (4*′*)-Ia (3)* were common. All the MRSA isolates were biofilm producers, whereas in MSSA isolates half of the isolates were able to produce biofilm. The results indicated that adhesion genes *clfA* and *clfB* were detected in all of the MRSA strains, and the most common *ica* genes were *icaA* and *icaB*. All the MSSA isolates carried *clfA*, *clfB*, and *fnbB* simultaneously, and *icaA* and *icaD* were the most prevalent biofilm-related genes. The isolates identified as CC22/ST22-MRSA-IV isolates showed a prevalence of 37.5% (3/8) for the *pvl* and *tst* genes. One isolate carried *sea* gene (12.5%, 1/8). The *sec* gene (30%, 3/10) was the only toxin gene detected among CC22/ST22-MSSA isolates. The carriage of *pvl* genes was more common among MRSA isolates than among MSSA isolates. The SGB + SGF + SGFa + SGFb prophage profile was detected in the majority of CC22 isolates.

#### CC30

CC/ST30 isolates were found in both MRSA and MSSA isolates. Among MSRA isolates, the most predominant *spa* types were t605 and t019, representing 40% (2/5) and 60% (3/5) of isolates. Multiple resistance genes including *erm*(C), *aac (6*′*)-Ie/aph (2*′′), *aph (3*′*)-IIIa*, and *tet*(M) were identified among tested isolates. More than half of the CC/ST30-MRSA-IV isolates carried *pvl*, whereas isolates harboring *sea* were at a relatively low frequency. All CC/ST30-MRSA-IV isolates displayed agr type III and *coa* type II. Four isolates (80%) were able to produce biofilm, and the remaining 20% were non–biofilm producers. All the MRSA isolates carried biofilm (*icaA*, *icaB*, *icaC*, and *icaD*) and adhesion (*clfA*, *clfB*, and *fnbB*) related genes. In all the MSSA strains, *fnbA*, *cna*, and *icaA* genes were detected. Among MSSA isolates, *spa* types t021 and t318 represented 70% (7/10) and 30% (3/10) of isolates. Nearly 90% (9/10) of the CC/ST30-MSSA isolates could form biofilm at different intensities. Various prophage patterns with the majority of SGB (60%, 6/10) were identified among MSSA isolates. The majority of CC/ST30-MSSA isolates exhibited erythromycin resistance encoded by *erm*(B) (80%, 8/10), and *erm*(C) (50%, 5/10). The *pvl* and *sed* encoding genes were the only toxins produced by this genotype. The SGA + SGB + SGF + SGFa + SGFb + SGL was found as the most predominant prophage profile among CC/ST30-MRSA-IV isolates.

#### CC45

All ST45-MRSA-IV isolates were assigned a *spa* type t038, *agr* I, and *coa* V. The isolates that exhibited resistance to multiple antibiotics were confirmed among these isolates. More than half of them (60%) were biofilm producers. All of these isolates carried *icaB*, *clfA*, *clfB*, and *fnbB* encoding genes simultaneously. All ST45-MRSA-IV isolates possessed *erm*(A) gene. The carriage of *aac* (6′)*-Ie/aph* (2′′) gene was found in 60% of isolates. The enterotoxin gene *sec* was present in 80% of isolates. Among prophage patterns, the SGF + SGFa + SGFb was common.

#### CC59

Two CC5 MRSA isolates were detected as one with ST59 and another one with ST338. All CC59 isolates indicated a single *spa* type, t437, *coa* type VI, and SGB-SGF-SGFa-SGFb phage pattern. All CC59/ST59 isolates were discriminated into SCC*mec* types IV. Two-thirds of ST59-MRSA-IV isolates carried *seb* gene; however, no other toxin genes were identified. Three isolates accounting for half (50%) of the entire ST59-MRSA-IV isolates were non–biofilm producers, and only one isolate showed strong biofilm formation. Results also demonstrated that the *icaA*, *icaD*, *clfA*, and *clfB* genes were detected in all of the CC59 isolates. Almost one-fifth of these isolates were resistant to fusidic acid encoded by *fusB* gene. Four and two ST59-MRSA-IV isolates carried *erm*(C) (66.7%) and *erm*(B) (33.3%), respectively. All CC59/ST338 isolates were assigned into an SCC*mec* type III. Half of CC59/ST338-MRSA-III isolates harbored *see* encoding gene. Other toxin-encoding genes were not noticed. The predominant isolates carried *erm(B)* (75%, 3/4), and aminoglycoside resistance encoded by *aph (3*′*)-IIIa* was also common among these isolates (50%, 2/4). All of the CC59 isolates possessed SGB + SGF + SGFa + SGFb prophage profile.

#### CC80

The CC80 isolates exhibited different *spa* types including t044 (66.7%, 8/12), t131 (33.3%, 4/12). All the ST80-MRSA-IV isolates assigned *agr* III and *coa* II. One-fourth of isolates were categorized as non–biofilm producers. The results exhibited that the *clfA* and *clfB* genes were explored in the entire CC80 isolates. The gene encoding for *icaD* (91.7%, 11/12) was the most prevalent biofilm-related gene among isolates.

The carriage of *pvl* genes was confirmed in one-third of tested isolates. Resistances to aminoglycosides encoded by *ant* (4′)*-Ia* (41.7) and *aac (6*′*)-Ie/aph (2*′′) (33.3), erythromycin encoded by *erm*(A), and fusidic acid encoded by *fusB* were common. The most prevalent prophage pattern was SGA + SGB + SGF + SGFa + SGFb + SGL.

## Discussion

This cross-sectional study provided several novel findings regarding the prevalence and genetic diversity of iMLSB-positive *S. aureus* strains isolated from clinical samples. It was observed that there was approximately a threefold increase in the prevalence of iMLSB *S. aureus* strains, from 7.5 to 21.7%, between 2013 and 2018. In contrast, Chavez-Bueno et al. (2005) suggested a significant decline in the trend for the prevalence of iMLSB phenotype from 93% in 1999 to 7% in 2002. Although our results markedly exhibited an increase in the prevalence of the inducible resistance among clinical *S. aureus* isolates in Iran, enhanced clinical and laboratory awareness of inducible resistance may also be responsible for this rise. Molecular epidemiology and prevalence of inducible resistance among *S. aureus* strains change geographically and dynamically (Monecke et al., 2011; Boswihi et al., 2016). In this experiment, the prevalence of iMLSB among *S. aureus* was found to be 10.9% with 84.1% MRSA and 15.9% MSSA isolates exhibiting iMLSB. This prevalence was lower than the rates reported from Nepal (21%) (Adhikari et al., 2017) and Jordan (76.7%) (Aqel et al., 2017), whereas it was higher than Brazil (7.9%) (Bottega et al., 2014). However, variable rates of iMLSB among *S. aureus* isolates have been reported in previously published data from Iran ranging from 4.1 to 20.7% (Memariani et al., 2009; Khashei et al., 2018). Regarding the increasing incidence of inducible resistance, our study suggests the necessity to revise the prescription of macrolides, which could cause a decline in resistance patterns. The investigated population and the prevalence of different clones in across various regions of the world could likely explain the striking differences in inducible clindamycin resistance.

In the present experiment, 18.3% of isolates tested were found to be resistant to mupirocin. This prevalence is higher than that reported rate in Jordan (5%) (Aqel et al., 2012) and other reports in France (2.2%) (Desroches et al., 2013) and Greece (1.6%) (Petinaki et al., 2004). A research performed by Abbasi-Montazeri et al. (2013) in Iran notified that 6% of *S. aureus* strains obtained from burn patients were detected as mupirocin-resistant. A high prevalence of resistance to mupirocin in this experiment indicates that there are unrestricted policies in the use of mupirocin for long periods and/or increasing trends of mupirocin prescription in our setup. Furthermore, we observed that 9.6 and 8.7% isolates had HLMUPR and LLMUPR phenotypes, respectively, which are higher than the rate stated in France (0.8%) (Desroches et al., 2013) and Korea (5%) (Yun et al., 2003). Different prevalence rates of HLMUPR *S. aureus* were reported from other researchers in Iran, 25% by Shahsavan et al. (2012) and 17% by Abbasi-Montazeri et al. (2013). We found that 9.5% of the examined isolates carried *mupA* gene, and all of them exhibited an HLMUPR phenotype. Researches from Spain (González-Domínguez et al., 2016) (27.2%) and Iran (Abbasi-Montazeri et al., 2013) (34%) revealed a higher rate of this gene among their *S. aureus* strains. Contrary to the study of Shahsavan et al. (2012), reporting *mupA* gene only in MRSA strains with cMLSB phenotype, the present data showed the existence of *mupA* gene in inducible resistance MRSA strains.

This study indicated that of 126 inducible clindamycin-resistant *S. aureus* isolates, 5.6% were fusidic acid–resistant, which was similar to those in Canada (7%) and Australia (7%); nonetheless, it was far lower than the values previously reported in Greece (62.4%) and Ireland (19.9%) (Castanheira et al., 2010a, b). In a multicenter experiment performed from 2007 to 2011 in three referral hospitals in Tehran, Iran, of 726 tested *S. aureus* isolates, 3% of isolates were found to be resistant to fusidic acid (Deotale et al., 2010). In our collections, we found that *fusB* and *fusC* were present in six strains and one strain, respectively. A previously studied experiment from China noted a higher rate of *fusB* gene (10.5%); on the other hand, *fusC* and *fusA* genes were not present in any of tested isolates (Yu et al., 2015). These data highlighted that *fusB* is the predominant determinant responsible for resistance to fusidic acid among *S. aureus* in Iran. According to previously published data, different fusidic acid resistance rates were reported in both MRSA and MSSA strains. Notably, this study showed that fusidic acid resistance was only seen among MRSA isolates, which were in line with a report of China indicating that the prevalence of fusidic acid resistance among MRSA isolates was significantly higher than that among MSSA isolates (Yu et al., 2015).

According to the evidence, the ability to produce biofilm among *S. aureus* is diverse, with data ranging within 43 to 88% (Luther et al., 2018). Our results showed that, of the 126 isolates under investigation, 77% of strains could produce biofilm, whereas 23% were confirmed as non–biofilm producer strains. This finding is similar to those in Egypt (83.3%) (Gad et al., 2009), whereas based on a study of Iran biofilm production reported rate was 38.7% (a twofold decrease) (Mirzaee et al., 2014). In this study, the biofilm formability of MRSA isolates (78.3%) was higher than that of MSSA isolates (70%). Apparently, high capability of biofilm formation among MRSA strains was described by conducted studies from India (57.6%) (Mathur et al., 2006), China (66%) (Wang et al., 2010), and South Africa (37.8%) (Samie and Shivambu, 2011).

Data obtained from different studies suggest that the ability of biofilm formation in *S. aureus* strains is attributed to the expression of a wide range of virulence and adhesion factors of this bacterium (Nemati et al., 2009; Samie and Shivambu, 2011; Luther et al., 2018), but the correlation between the existence of specific virulence factor and biofilm production has been controversial. Researches has shown that *ica ABCD* and adhesion genes can affect the biofilm formation ability in *S. aureus.* In this study, the most frequent *ica* genes were *icaD* (73%), followed by *icaA* (71.4%), *icaB* (60.3%), and *icaC* (53.2%). According to an analysis by Mirzaee et al. (2014) on 31 clinical *S. aureus* isolates, *icaD*, *icaA*, and *icaC* genes with the exception of *icaB* were found in more than half of the isolates tested. In the study of Yousefi et al. (2016), of 39 isolates of *S. aureus* recovered from UTI patients, 69.2% were biofilm producers, and it was notable that all isolates carried *icaA*, *fnbA*, and *clfA* genes. In the current study, a high percentage of *icaABCD* genes made up 39.7% of the overall sample, which was close to a recent report in Iran (38.7%) (Mirzaee et al., 2014). The correlation between biofilm formation and the presence of adhesion genes is well established. In present survey, the most prevalent gene was *clfA* (93.7%), followed by *clfB* (87.3), *fnbB* (61.9%), *cna* (57.9%), *fnbA* (54%), *ebp* (50%), and *bap* (3.2%), respectively. The attained data are consistent with a previous finding from Iran, which has displayed the role of the *bap* gene in biofilm production rarely. Our findings indicated that isolates with more adhesion and *ica* encoding genes were strictly associated with biofilm formation. Overall, our data confirmed the high ability of biofilm formation among inducible resistance *S. aureus* strains, which helps *S. aureus* to persist in infections. This scenario draws attention from clinicians to use treatment protocols in patients potentially infected with these bacteria.

Based on the results of *coa* typing, the top three *coa* types were III (44.4%), II (28.6%), and IVa (9.5%). This result was in contrast with the previous report by Hirose et al. (2010) in Japan, which indicated that *coa* types II, VII, and I accounted for 91.9%, 3.9%, and 1.7% of isolates. We detected seven SC types (I–VI) among iMLSB *S. aureus* strains suggesting diverse genetic backgrounds of tested isolates in this region of Iran. In a research involving 157 *S. aureus* strains from clinical specimens, nine different patterns of *coa* gene were detected (Afrough et al., 2013). These findings were confirmed by results in Thailand (Janwithayanuchit et al., 2006) and Egypt (Omar et al., 2014) reported previously. Genetic variability in *coa* gene among studied isolates indicated that it could not be a predictor for specific iMLSB *S. aureus* strains.

In the present study, the findings revealed that seven different prophage profiles were identified in the isolates studied. A high diversity of prophage patterns among *S. aureus* isolates has been reported from the United States (Workman et al., 2006), Czech Republic (Pantůček et al., 2004) and Iran (Rahimi et al., 2012). Our data exhibited that SGFa-SGFb (36.5%) and SGB-SGFa-SGFb (31.7%) were the major prophage profiles, which was in accordance with the study of Rahimi et al. (2012). SGF-type prophages are associated with the immune evasion cluster typical for *S. aureus* isolated from humans (Kahánková et al., 2010). SGA has been reported to be common among *pvl*-positive MRSA-IV isolates. This finding was consistent with previous research, revealing that all the *pvl-*positive isolates harbored SGA prophage type (Rahimi et al., 2012). Notably, the PVL was the most frequently encoded toxin in the tested isolates (23.8%). Previously published data from England and the United States (Holmes et al., 2005) indicated that the prevalence of *pvl* encoding genes among *S. aureus* was low (1.6%). Nevertheless, previous reports all over the world had shown a high prevalence of *pvl* among *S. aureus* strains (Monecke et al., 2011; Boswihi et al., 2016; Goudarzi et al., 2016a). In Iran, the *pvl*-positive rate ranged from 7.4 to 55.6% in *S. aureus* isolates. In our research, a relatively low but increasing prevalence of *pvl*-positive *S. aureus* strains was noted. According to our report, a recent study from Ireland showed an ascending trend of *pvl* among MRSA strains (from 0.2 to 8.8%), whereas this trend was diminishing for MSSA strains (20–2.5%) (Shore et al., 2014). Although this difference can, to some extent, reflect the origin of isolates and the type of sample, this could also be because of different *pvl*-encoding phages among *S. aureus* strains.

Considering the genes encoding enterotoxins, the data analysis of the current study exhibited that 54.8% of isolates carried one or more SE genes, with the *sea* (19.8%) and *sec* (11.1%) genes being the most commonly found. The attained data are consistent with the reports previously obtained from Iran and Turkey estimating enterotoxigenic *S. aureus* strains in 45 and 62.6% of isolates tested, respectively (Imanifooladi et al., 2007; Aydin et al., 2011). Based on earlier studies, the *she*, *sei*, and *sej* encoding genes were rarely present in *S. aureus* isolated from clinical samples, and similarly, these genes were not detected in the present survey either.

The present study displayed diversity in the numbers and the molecular types of iMLSB *S. aureus* clones identified in our health care settings. According to the current results, CC8 was the most prevalent clone in both CO and HO *S. aureus* isolates. The prevalence of CC8 was increased from 2013–2014 (20%) to 2017–2018 (39.5%). These strains were found in association with *pvl-*encoding bacteriophage and HLMUPR, which carried *mupA* gene, which is similar to those reported in Iran (Azimian et al., 2014), Kuwait (Boswihi et al., 2016), and Ireland (Shore et al., 2014). According to evidence, phenotypic and genotypic resistance pattern in the ST8-IV isolates was found to be varied. In accordance with our research, high resistance to aminoglycosides and low resistance to tetracycline in ST8-MRSA isolates were described previously by researchers (Shore et al., 2014; Boswihi et al., 2016). The enterotoxin gene *sed* was the solo toxin gene detected among the ST8-IV isolates. Variability in the enterotoxin genes within ST8-IV isolates was reported by several investigators (Shore et al., 2014; Boswihi et al., 2016). The detection of the ST8-IV isolates in our study is of concern and highlights transmissibility and rapidity of its spread as an international epidemic MRSA clone in Iran.

ST239-MRSA-III, one of the most successful and persistent clones, was the most predominant genotype in CC8. Multiresistant ST239 clone was previously reported in South American, European, and some Asian countries, including Kuwait and Saudi Arabia (Shore et al., 2014; Boswihi et al., 2016). In this study, ST239 had five important *spa* types (t030, t037, t631, t860, and 388). The data in this experiment indicated that half of ST239-SCC*mec* III/t631 strains exhibited HLMUPR phenotype and harbored *mupA* gene. These findings are in parallel with previous reports from Iran (Goudarzi et al., 2016a) and those reported in India and Kuwait (Abimanyu et al., 2012; Boswihi et al., 2016). All ST239-SCC*mec* III/t860 strains exhibited the LLMUPR phenotype, which was similar to findings in other countries including China and Kuwait (Boswihi et al., 2016; Liang et al., 2018). According to the molecular typing results, ST239-SCC*mec* III/030 with 20.5% and ST239-SCC*mec* III/037 with 25% were recognized as the most common multiresistant ST239- MRSA-III type. These results were consistent with previous research performed by Li et al. (2018), who demonstrated multiresistant ST239-SCC*mec* III/030 as the most predominant genotype in China from 2005 to 2013, which decreased significantly in 2016. During a 12-year period in Norway, Fossum and Bukholm (2006) indicated ST239 as the most prevalent MRSA clones, which became endemic in hospital environments in Norway. However, molecular characteristics, especially antibiotic resistance gene and virulence factors, of our ST239-MRSA-III strains were similar to those of ST239 strains reported in Kuwait, China, and Saudi Arabia (Lewis and Jorgensen, 2005; Boswihi et al., 2016). The emergence of ST239-MRSA-III may be due to the import of this clone from neighboring countries.

Increasing prevalence rate of CC8 and disappearance of CC22 between 2013 and 2018 highlighted replacement and the changing clonal structure of MRSA in this region of Iran. As summarized in Table 5, *pvl* encoding genes were detected in two isolates of ST22-SCC*mec* IV/t852 and one isolate of ST22-SCC*mec* IV/t005. These *pvl*-positive genotypes were mainly distributed at some geographic area including England, Australia, Ireland, Kuwait, Iran, Germany, Saudi Arabia, and Nepal (Monecke et al., 2011; Shore et al., 2014; Boswihi et al., 2016). Of note, the majority of the ST22-SCC*mec* IV/t790 isolates was related to the *tst* gene, demonstrated LLMUPR phenotype, and could exhibit multiresistance. A recent report in Ireland also displayed that most of *pvl*-positive ST22-MRSA-IV isolates were associated with resistance to gentamicin, trimethoprim, and ciprofloxacin and carried the *erm(C)*, *lnu(A)*, *aacA-aphD*, *aadD*, and *mupA* genes (Shore et al., 2014).

The ST22-MSSA isolates exhibited different antimicrobial resistance patterns including resistance to penicillin, gentamicin, tetracycline, kanamycin, rifampin, tobramycin, and amikacin. Our data are in concordance with a study conducted in Ireland, which reported the prevalence of low frequency of ST22-MSSA/t005 and ST22-MSSA/t1869 strains during the study period from 2002 to 2011. They indicated that ST22-MSSA was resistant to amikacin, ampicillin, fusidic acid, gentamycin, kanamycin, and tobramycin with multiple resistance genes *blaZ*, *aacA-aphD*, and *dfrS1* detected among these isolates (Shore et al., 2014).

Our data showed that 40% of the CC/ST30 isolates carried *pvl*, which belonged to MRSA and MSSA strains. PVL-positive CC30/ST30-IV strains have been described in Iran (Goudarzi et al., 2016a, b), Kuwait (Boswihi et al., 2016), and Ireland (Shore et al., 2014). Importantly, the prevalence of CC30/ST30 increased from 8% in 2013 to 18.4% in 2018 in our country. In this connection, Boswihi et al. exhibited ST30 as the second dominant MRSA genotypes in Kuwait hospitals, which decreased from 30% in 2001–2003 to 22% in 2006. They also showed a low prevalence of ST30-IV-MRSA among tested isolates (2.9%) (Boswihi et al., 2016). Surprisingly, all MRSA isolates carried biofilm and adhesion-related genes, and approximately 90% of the MSSA isolates were able to form biofilm at different intensities. In line with our study, Chamon et al. (2015) from Brazil showed biofilm production ability among ST30 strains. They also found *bbp* gene as a possible marker of this lineage. Congruent with the previous observations (Shore et al., 2014; Boswihi et al., 2016), all of our ST30 isolates belonged to *agr* type III, with different toxin and antimicrobial resistance patterns noted for this genotype.

As aforementioned, fewer than half of the CC/ST80-MRSA-IV isolates showed aminoglycoside resistance encoded by *ant* (4′)*-Ia*, *aac (6*′*)-Ie/aph (2*′′). Resistance to fusidic acid encoded by *fusB* was detected in one isolate. These results were different from a previous study conducted in Ireland, which exhibited all CC/ST80-MRSA-IV isolates were resistant to kanamycin and neomycin, encoded by *aph (3*′*)-IIIa*. In addition, our data revealed that resistances to tetracycline, fusidic acid, and erythromycin encoded by *tet(K)*, *fusB*, and *erm*(C), respectively, were frequent among tested isolates (Shore et al., 2014). The variations could be a result of differences in the genetic backgrounds of the *S. aureus* strains. The prevalence of CC/ST80-MRSA-IV increased from 12% in 2013 to 13.2% in 2018. This increasing rate of ST80-IV raises the concern that this strain is becoming endemic in our hospitals.

Based on the evidence, ST80-IV is acknowledged as a toxigenic virulent isolate. In the present study, *pvl*-positive CC/ST80-SCC*mec*/t044 isolates were confirmed by approximately half of the isolates. ST80-MRSA-MRSA isolates harboring the *pvl* genes were previously reported from Kuwait, Malaysia, Singapore, and Ireland (Shore et al., 2014; Boswihi et al., 2016).

According to previously published data, CC/ST59 has limited geographical spread. In the present research CC/ST59 was present in 10 isolates, accounting for 7.9%. This clone was previously reported in Australia, Ireland, United Kingdom, Korea, Kuwait, and Taiwan (Monecke et al., 2011; Boswihi et al., 2016). In this study, the results revealed that our isolates carried resistance genes at a relatively low level, but *erm*(C) encoding resistance to erythromycin was noted in more than half of the isolates. Conversely, high frequencies of multiple resistance genes including *erm*(B), *aph (3*′*)-IIIa*, and *tet*(K) were identified among tested isolates of a study performed in Ireland (Shore et al., 2014). Notably, resistance to fusidic acid encoded by *fusB* was also detected in one isolate, which was similar to the previous report from Ireland (Shore et al., 2014). According to this experiment, 66.7% of the isolates were positive for *seb* (60.4%) gene. This is inconsistent with the research conducted in Ireland showing that *seb* gene could be a possible marker of this lineage. The observed frequency of CC59/ST338-SCC*mec*III/t437 isolates was in accordance with previous reports, as this genotype is infrequently isolated (Li et al., 2013).

Another clone found among inducible resistance *S. aureus* strains was ST5-SCC*mec* IV/t002 (7.1%). Recent studies have shown the presence of CC/ST5- SCC*mec*IV/t002 clones in Asian and European countries, such as Iran, Japan, Korea, the United Arab Emirates, Kuwait, Ireland, and Australia (Monecke et al., 2011; Boswihi et al., 2016). The present data indicated that the prevalence of CC/ST5- SCC*mec*IV/t002 diminished during the study from 8% in 2013–2014 to 2.6% in 2017–2018. Conversely, a recent cross-sectional study performed in New Zealand on 3,323 patients from 2005 to 2011 documented seven most frequent MRSA clones. This study also indicated an ST5-SCC*mec*IV clone, which rapidly displaced ST30-SCC*mec*IV as the dominant CA-MRSA clone (Williamson et al., 2013). It was also observed that nearly half of ST5-SCC*mec*IV/t002 isolates were confirmed as HLMUPR strains. The finding of CC/ST5- SCC*mec*IV/t002 isolates in our screening indicated that resistance was observed for erythromycin encoded by *erm*(A), tetracycline encoded by *tet*(M), and mupirocin encoded by *mupA*. In an experiment conducted in 2010 in China, Song et al. (2013) revealed a different result. They showed a high prevalence of ST5-SCC*mec*IV/t002 among their clinical *S. aureus* isolates.

As shown in Table 5, the CC1 isolates belonged to two STs (ST772 and ST1). *spa* types t10795 and t657 pertained to ST772, whereas *spa* type t6811 was identified in ST1 isolates. ST772- SCC*mec*V, which is known as Bengal Bay clone, emerged in Bangladesh and was reported in New Zealand, Nepal, Italy, the United Arab Emirates, Malaysia, the United Kingdom, Ireland, Saudi Arabia, India, Australia, and Kuwait (Monecke et al., 2011; Shore et al., 2014; Boswihi et al., 2016). This clone was found in 6.4% of the examined isolates. All isolates were multiresistant and carried *aac (6*′*)-Ie/aph (2*″), *aph (3*′*)-IIIa*, *tet*(M), and *erm(B)*. The attained data are in accordance to results of the study of Shore et al. (2014), which reported MDR pattern and carriage of *ant (4*′*)-Ia*, *aac (6*′*)-Ie/aph (2*″), and *tet*(M) genes among CC1/ST772-SCC*mec*V isolates. The study demonstrated similarities in genetic characteristics including susceptibility to antibiotics and toxin-encoding genes of our ST772-SCC*mec*V isolates recently reported in Kuwait (Boswihi et al., 2016). Of two CC1/ST1-SCC*mec*V/t6811 isolates, one isolate carried *fusc* and exhibited resistance to fusidic acid at MIC 32 μg/mL. Boswihi et al. (2016) previously reported ST1-SCCmecV/t6811 carrying *fusC* gene from Kuwait.

In contrast to our study, which reported a low prevalence of ST15 (4%), a recent multicenter study performed in 25 European countries documented a relatively high prevalence of ST15 and reported it as the second most frequent clone across most of the European countries (Grundmann et al., 2014). In a study of 568 *S. aureus* isolates in 11 European countries, researchers reported a low level of this type among the tested isolates (Rolo et al., 2012). Our data demonstrated that all the ST15 isolates were *pvl-*positive and displayed the LLMUPR phenotype. The analysis of our previous study indicated that the most common mupirocin-resistant MRSA isolates belonged to ST15-SCC*mec* IV/t084 (Goudarzi et al., 2017), which is in line with the present data. Although ST15 is more frequently detected in MRSA, there are reports that indicate the high distribution of this type among MSSA strains (Monecke et al., 2011; Boswihi et al., 2016). It was notable that all ST15 isolates harbored SCC*mec* IV, *agr* type II, and *coa* type I and belonged to *spa* type t084. Different antimicrobial resistance patterns were noted among these isolates, which is in accordance with previous studies (Rolo et al., 2012; Grundmann et al., 2014; Goudarzi et al., 2017).

In contrast to a study conducted in Kuwait, which reported CC45 as one of the most epidemics MRSA isolates with different antimicrobial resistance patterns, in the current research, a low frequency of the CC45 (4%) was observed among examined isolates. In a study conducted in the South of Poland, 26.1% of *S. aureus* isolates were found to be related to CC45 (Ilczyszyn et al., 2016). This ST was at its most prevalent in 2015, when it accounted for 22.2% (4/18) of inducible resistance isolates, reduced to 4.2% (1/24) in 2016, and disappeared in 2017–2018.

The strengths of our research included examining the prevalence and temporal differences in iMLSB *S. aureus* strains. It was the first study on the molecular characterization of inducible clindamycin-resistant *S. aureus* strains from Iran. However, the present research had limitations. One limitation of our study was the modest sample size and the impossibility of using typing methods such as pulsed-field gel electrophoresis (PFGE). It was not possible either to correlate demographics data with circulating clones owing to a lack of data linking patient characteristics. Another important limitation of our study was *clfA*- and *clfB*-negative *S. aureus* strains, which were unusual and required further analysis using whole-genome sequencing and microarray system.

## Conclusion

This was the first report of monitoring the prevalence and characterization of *S. aureus* isolates with the inducible resistance phenotype in Iran. Our investigation supports a detailed epidemiological survey on the prevalence and temporal differences in iMLSB *S. aureus* strains. It was ultimately attained that there is a considerable increasing trend for CC8 versus a decreasing trend for CC22. However, we revealed a shift in the clonal composition of MRSA isolates over time with the emphasis on a progressive replacement of CC22 clone by CC8 clones between 2013 and 2018. Indeed, our research indicated that iMLSB *S. aureus* isolates with similar genetic backgrounds exhibited specific virulence gene profiles, antimicrobial resistance patterns, and biofilm patterns. Increase in *S. aureus* with inducible resistance phenotype harboring SCC*mec*IV during the 5-year period makes sense that there is a shift in the iMLSB population from our community to hospital. Therefore, we conclude that there is a need for ongoing and nationwide surveillance studies to further evaluate of *S. aureus* with inducible resistance phenotype and to prevent these strains from becoming endemic in the Iranian hospitals.

## Data Availability Statement

The datasets generated for this study are available on request to the corresponding author.

## Ethics Statement

The current study protocol was approved by the Ethics Committee of the Shahid Beheshti University of Medical Sciences in Tehran, Iran (IR.SBMU.MSP.REC.1396.700). Written informed consent was obtained from participants.

## Author Contributions

MG and HG conceived, designed, and supervised the study. MD, RaP, MN, MF, MM, SS, and AA performed the experiments. NK, RoP, and MG analyzed and interpreted the data. MG, HG, NK, SS, and RoP drafted and written the manuscript. All authors approved the final version of manuscript.

## Conflict of Interest

The authors declare that the research was conducted in the absence of any commercial or financial relationships that could be construed as a potential conflict of interest.

## Footnotes

1 http://www.spaserver.ridom.de

2 https://pubmlst.org/
